# Social Intercourse

**Published:** 1870-12

**Authors:** 


### SOCIAL INTERCOURSE.

The material "progress" of the world has brought the most distant nations to our doors, and put rivers, and mountains, and seas, between friends and neighbors. All of us feel that there is far less social intercourse between families than there was a score or two of years ago; the essential reason of which is, it requires so much more effort to keep our houses and ourselves in a presentible shape, that we have not time to make an old-fashioned visit; that is, to go and see a neighbor before sun-down, and stay and take tea, and then laugh and talk to a late hour in the night, reaching home with pleasant memories of the good cheer, the well-spread table, and the vigorous appetite for its consumption. All thoughtful minds should cultivate social intercourse as a matter of principle, and pleasure, and duty; it breaks up the monotony of domestic life, it promotes that interchange of ideas, and that reciprocity of courtesies which cherishes self-respect, which wakes up those ambitions and commendable rivalries which are calculated to elevate the individual in particular, and society in general.

Our farmers, especially, work too hard in the daytime, and are too tired, when evening comes, to dress and go and see a neighbor; hence, there is a monotony in their existence which allows the faculties to go to sleep; and to this monotony they soon become so accustomed that they become in time but little more than machines, have not the energy requisite to take them out of one treadmill course, and they go round and round the same beaten track until they die, and too often but little above the elevation of mind which belongs to the brute creation.

